# MicroRNA-Mediated Metabolic Shaping of the Tumor Microenvironment

**DOI:** 10.3390/cancers13010127

**Published:** 2021-01-03

**Authors:** Federico Virga, Lorena Quirico, Stefania Cucinelli, Massimiliano Mazzone, Daniela Taverna, Francesca Orso

**Affiliations:** 1 Molecular Biotechnology Center (MBC), University of Torino, 10126 Torino, Italy; federico.virga@edu.unito.it (F.V.); lorena.quirico@unito.it (L.Q.); stefania.cucinell@edu.unito.it (S.C.); massimiliano.mazzone@kuleuven.vib.be (M.M.); daniela.taverna@unito.it (D.T.); 2 Department Molecular Biotechnology and Health Sciences, University of Torino, 10126 Torino, Italy; 3 VIB Center for Cancer Biology (CCB), Department of Oncology, University of Leuven, B-3000 Leuven, Belgium

**Keywords:** miR, metabolism, tumor microenvironment

## Abstract

**Simple Summary:**

Ongoing studies demonstrate the importance of metabolism in cancer development and progression. Metabolic alterations occur, not only in cancer cells, but also in the tumor microenvironment (TME). The continuous crosstalk between tumor cells and stromal and immune cells deeply affects metabolic rewiring. miRs, small noncoding RNAs involved in post-transcriptional regulation, are important mediators in these processes. In-depth knowledge of these interactions is crucial in understanding cancer progression and, consequently, for the development of new therapies.

**Abstract:**

The metabolism of cancer cells is generally very different from what is found in normal counterparts. However, in a tumor mass, the continuous crosstalk and competition for nutrients and oxygen among different cells lead to metabolic alterations, not only in cancer cells, but also in the different stromal and immune cells of the tumor microenvironment (TME), which are highly relevant for tumor progression. MicroRNAs (miRs) are small non-coding RNAs that silence their mRNA targets post-transcriptionally and are involved in numerous physiological cell functions as well as in the adaptation to stress situations. Importantly, miRs can also be released via extracellular vesicles (EVs) and, consequently, take part in the bidirectional communication between tumor and surrounding cells under stress conditions. Certain miRs are abundantly expressed in stromal and immune cells where they can regulate various metabolic pathways by directly suppressing enzymes or transporters as well as by controlling important regulators (such as transcription factors) of metabolic processes. In this review, we discuss how miRs can induce metabolic reprogramming in stromal (fibroblasts and adipocytes) and immune (macrophages and T cells) cells and, in turn, how the biology of the different cells present in the TME is able to change. Finally, we debate the rebound of miR-dependent metabolic alterations on tumor progression and their implications for cancer management.

## 1. Introduction

Tumors arise from normal cells that progressively evolve from an uncontrolled proliferating neoplastic mass to a malignant state where cells start to migrate invading the surrounding tissue [1]. Lately, malignant cells disseminate and spread in the body through the blood or lymphatic system causing distant organ metastases that are responsible for almost 90% of cancer-related deaths [2]. The International Agency for Research on Cancer estimated 18.1 million new cases and 9.6 million cancer-related deaths worldwide in 2018. Lung, female breast, and colorectal cancers account for one-third of global incidence and are also among the five most fatal types of cancer. Due to the rise in life expectancy and the increased presence of risk factors, cancer incidence and mortality are rapidly growing [3].

Uncontrolled proliferation, cell death resistance, replicative immortality, growth suppressor evasion, angiogenesis induction, invasion, and metastasis activation are all considered hallmarks of cancer [1]. Eleven years after the first edition of *The Hallmarks of Cancer*, metabolic energy reprogramming and immune escape avoidance mechanism have been highlighted and added as the emerging new hallmarks of cancer together with tumor-promoting inflammation, genome instability and mutations [4]. This underlines how our current understanding of cancer biology has shifted from a “cancer cell centric” perspective to a more inclusive concept which places cancer cells in a complex and interconnected network composed by the extracellular matrix (ECM), immune and stromal cells to which we generally refer as tumor microenvironment (TME) [5]. TME can collaborate with cancer cells promoting tumor progression and metastatization as well as resistance to therapy [6].

The ECM consists of various proteins including collagen, proteoglycans, glycoproteins, laminin, and fibronectin forming supramolecular aggregates. In the presence of a neoplastic disease, the ECM is in continuous remodeling. In fact, cancer cells are able to degrade proteolytically the ECM and infiltrate it. Furthermore, soluble factors secreted by cancer or stromal cells are able to dictate the formation of an advantageous ECM in distant organs, rendering engraftment of upcoming cancer cells and the establishment of metastasis possible. 

**Figure 1:**
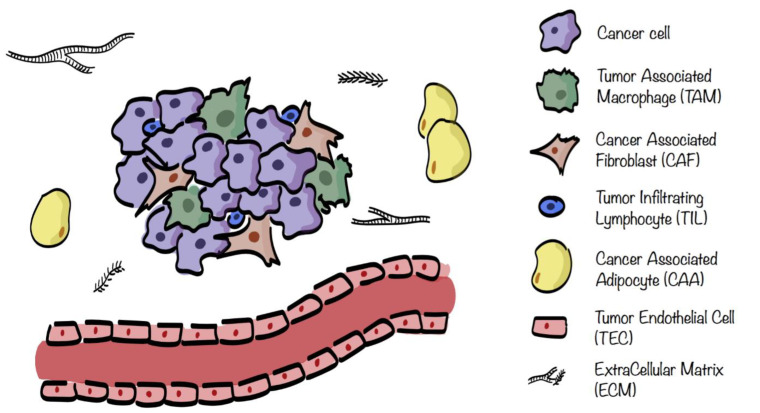
Tumor microenvironment (TME) composition. TME surrounding cancer cells consists of extracellular matrix (ECM) in which are embedded several stromal cells such as cancer associated fibroblasts (CAFs), tumor endothelial cells (TECs), cancer associated adipocytes (CAAs), and infiltrating immune cells (ICs), including tumorassociated macrophages (TAMs) and tumor infiltrating lymphocytes (TILs).

The different stromal cells of the TME are cancer associated fibroblasts (CAFs), tumor endothelial cells (TECs), mesenchymal stem cells (MSCs), cancerassociated adipocytes (CAAs), and infiltrating immune cells (ICs), including tumorassociated macrophages (TAMs), natural killers (NKs), neutrophils, dendritic cells (DCs), and tumor infiltrating lymphocytes (TILs), see Figure 1. These cells are characterized by specific metabolic traits and pathways that, depending on the surrounding situation, get modulated not only in cancer cells, but also within the TME [4,7]. 

An in-depth knowledge of the molecular mechanisms involved in the regulation of the metabolic crosstalk between cancer and non-malignant cells present in the TME and how stromal cells rewire their metabolism may pave the way for new therapeutic breakthroughs. In the present review, we examine recent insights into how miRs mediate this metabolic shaping of the TME by repressing metabolic enzymes, transporters, and other metabolic regulators.

## 2. Cancer Metabolism

The Nobel laureate Warburg one century ago made the pivotal observation that cancer cells rely more on glycolysis even in the presence of oxygen, a condition known as “aerobic glycolysis” or– “Warburg effect”, anticipating by many decades the discovery of oncogenes [8]. Despite this, metabolism was not mentioned in the first edition [1] and was only added in the later issue of *The Hallmarks of Cancer* [4], highlighting a growing interest in tumor metabolism. In addition to Warburg’s work, several investigations showed that in order to support their proliferation, cancer cells engage aerobic glycolysis [9]. Although the oxidative phosphorylation (OXPHOS) is more efficient in generating ATP, the glycolytic intermediates can be diverted to support the biosynthesis of macromolecules needed for proliferation [10]. Moreover, cancer cells compensate the inefficient ATP production by enhancing glucose uptake and its consumption [11]. More recently, metabolic intra-tumoral heterogeneity and metabolic crosstalk within different areas of the tumor mass have been suggested and characterized [12,13,14]. For example, Sonveaux et al. introduced a model of metabolic symbiosis between well-oxygenated and hypoxic regions of the tumor, based on lactate exchange [15]. Specifically, hypoxic cells engage anaerobic glycolysis releasing lactate which, in turn, is uptaken, instead of glucose, and used by oxygenated cancer cells to support their OXPHOS. A similar mechanism of metabolic symbiosis has been linked to resistance to anti-angiogenic therapies [16,17,18], indicating the relevance of cancer metabolism for cancer response to treatments. Due to metabolism plasticity and redundancy, targeting metabolism is challenging. An interesting approach to overcome this issue is represented by the depletion of the voltage-dependent anion channel 1, VDAC-1. Loss of VDAC-1 impairs cancer cell energy and metabolic homeostasis via the activation of a complex transcriptional program associated with metabolic regulation [19].

Recently, miRs have been found to regulate metabolic reprogramming as well. For example, key glycolytic enzymes and glucose transporters (GLUTs) have been shown to be controlled by miRs, resulting in high glucose uptake and accelerated metabolism of cancer cells [20,21]. 

## 3. Tumor Microenvironment Metabolism

Cancer progression is sustained by the contribution of several non-malignant cells that are shaped and, which, in turn, influence cancer cell behavior and TME metabolic landscape [4,22]. For example, following the interaction with CAFs, cancer cells improve their invasiveness, their ability to intra- or extravasate as well as their stemness potential. All these processes create a stiff fibrotic matrix where the development of blood vessels is impaired, leading to inefficient delivery of nutrients and clearance of waste products of cellular metabolism, together with the formation of hypoxic areas [23]. This hypoxic response causes an enhanced glycolytic activity in the tumor cells with an increased lactate deposition. Cancer cells crosstalk metabolically with stromal cells within the TME and, in 2009, a metabolic coupling between CAFs and surrounding cancer cells was proposed and named “reverse Warburg effect”. While in the classical Warburg effect cancer cells display aerobic glycolysis in order to sustain their proliferation, in the “reverse Warburg effect” the cells of the TME are highly glycolytic whereas cancer cells are not. More in detail, cancer cells induce aerobic glycolysis in surrounding CAFs which start to release pyruvate and lactate used directly by cancer cells to fuel their TriCarboxylic Acid (TCA) cycle and OXPHOS [24]. This reprogramming is due to an increased expression of GLUT-1 and monocarboxylate transporter-4 (MCT-4) involved in glucose uptake and lactate release, respectively [25]. The increased release of lactate in the microenvironment contributes to its acidification, which, in turn, leads to activation of the matrix metalloprotease-9 (MMP-9) and of the epithelial-to-mesenchymal transition (EMT) program promoting tumor progression [26,27]. However, the ‘reverse Warburg effect’ model does not apply to all CAF-cancer cell interactions. In fact, pancreatic and ovarian CAFs consume lactate and display low glycolytic activity [28,29]. Moreover, cancer associated stromal cells also reshape their metabolic landscape forcing the production of ketone bodies and glutamine and activating the mitophagy [30]. All these molecules could be exploited through OXPHOS, thus reprogramming cancer cells towards a respiratory metabolism. Furthermore, the production of pro-inflammatory cytokines by stromal cells can push cancer cells towards an OXPHOS phenotype. The key regulator of the pro-inflammatory stimuli is the NF-kB transcription factor, involved in the control of energy balance and metabolic adaptation to glucose starvation, by upregulating mitochondrial function [31]. Acidosis of interstitial space develops as an intrinsic consequence of the Warburg effect. Indeed, in normal conditions, the extracellular pH is around 7.4 and its levels decrease to a range of 6.7–7.1 in cancer [32]. The highly glycolytic activity of tumor cells results in both lowering of intracellular pH and lactate accumulation, which, in turn, leads to the inhibition of glycolysis itself. In order to avoid such inhibition, cancer cells upregulate MCTs and Na^+^H^+^ exchangers (NHE) or H^+^ ATPase pumps, devoted to lactate and protons export, respectively [33]. Moreover, the acidification of the TME reduces the response of the immune system against the tumor [34,35] and it is detrimental for drug delivery and efficacy [36,37]. This happens because at least one pH-titratable group is present in common drugs which are usually weak bases. As a result, at lower pH, drugs can be protonated, thus affecting their possibility to permeate through the cell membrane [32]. For all these reasons, acidic TME targeting started to be considered for tumor therapy. In this line, small-molecule inhibitors, nanotheranostic systems responsive to pH to deliver chemotherapeutic drugs specifically to acidic microenvironment and pH-sensitive biomaterials were developed [38,39,40]. An example is represented by mesoporous organosilica particles (MONs) able to deliver doxorubicin to low pH compartments [39]. In parallel, acidosis can mediate also the ‘immune escape’. It was demonstrated that T cells exposed to acidic environment show an increased threshold of activation and an enhanced expression of negative regulatory signals, namely CTLA4 and IFNɣR2 [41].

ICs display a strong interplay between immune response and metabolism. Following activation, immune cells start to proliferate and to produce effector molecules such as cytokines and cytotoxic granules. In order to fuel the higher biomass and the increased energy demands, activated immune cells rewire—and reprogram their metabolic pathways [42]. Interestingly, several studies have shown that limiting levels of glucose impair T cell effector activity which relies on glycolysis and, therefore, on glucose availability [43,44]. Even if the pivotal function of cell metabolism is to provide energy and substrates, it is important to underline that it also plays an essential role in instructing the phenotype and the differentiation of ICs influencing cancer outcome and therapy resistance [7,42]. Therefore, a good understanding of the metabolic processes of ICs and how they are regulated is essential to harness anti-immunity response and improve cancer survival. Besides glucose, also fatty acids and amino acids, such as glutamine, are importantly involved in immune cell fate and response [45,46].

Overall, the metabolic reprogramming of TME is strongly affected by hypoxia, which leads to an impairment of OXPHOS, an enhancement of glycolytic activity and production of mitochondrial reactive oxygen species (ROS). In particular, oxygen deprivation pushes cancer cells towards a more glycolytic phenotype in a hypoxia-inducible factor-1α (HIF-1α)-dependent manner. In turn, HIF-1α activity relies on redox, oxygen levels, PI3K/mTOR/Akt, and c-MYC-related pathways involved in tumor cell metabolism, growth and survival [25,47,48,49]. HIF-1α, in fact, induces the expression of pyruvate dehydrogenase Kinase 1 (PDK1), leading to decreased activity of the pyruvate dehydrogenase complex (PDC) with a consequent reduction of oxygen consumption and enhanced glycolysis [50,51]. Moreover, HIF-1α increases glucose uptake and causes a more efficient glycolytic breakdown due to the induction of GLUT-1 [52] and GLUT-3 expression [53]. The metabolic crosstalk among tumor and stromal cells is finely regulated and extracellular vesicles (EVs) are key messengers in this communication. The analysis of EVs involved in metabolic rewiring revealed the presence of proteins, lipids, different RNA species and metabolites. Among the various RNAs present in EVs, miRs exert a central role in the metabolic crosstalk by directly suppressing metabolic enzymes or transporters as well as by controlling important regulators of metabolic processes such as HIF-1α, PI3K/mTOR/Akt, and MYC pathways. In fact, miRs are recently emerging as regulators of the metabolic reprogramming in ICs and CAFs [54,55].

## 4. MicroRNAs (miRs)

Considering that at least 80% of the mammalian genome is transcribed and less than 3% of the sequences encodes for protein-coding genes, it is clear that the non-coding part of the genome can be relevantly involved in physiological and pathological programs [56]. In this context, miRs are the most studied class of non-coding RNAs in cancer. As the name suggests, miRs are small (20–22 nucleotides) RNA molecules that have emerged as negative regulators of gene expression which bind to the 3′-untranslated regions (3′-UTRs) of their target mRNAs, causing a block of translation and/or mRNA degradation. MiRs were first discovered in 1993, when lin-4 was demonstrated to control the developmental timing of larval *C. elegans* [57]. Nowadays, we know that miRs are widely present in plants and animals [58]. Overall, it is estimated that over 2000 miRs are present in the human genome and can control the activity of nearly 60% of the protein-coding genes [59,60,61,62].

**Figure 2:**
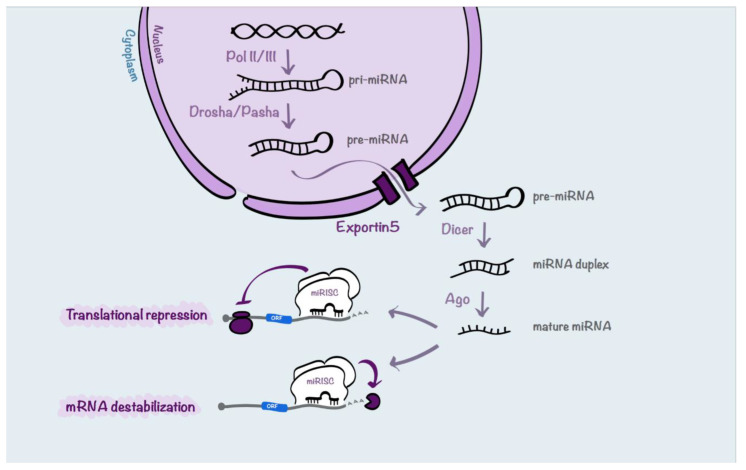
Schematic representation of miR processing. miRNA biogenesis begins with RNA polymerase II-dependent transcription that generates pri-miRNAs. Then, the pri-miRNAs are processed into pre-miRNAs by Drosha and Pasha. Next, pre-miRNAs are transported by Exportin5 from the nucleus to the cytoplasm where they are processed by Dicer into miRNA duplexes. The duplexes are loaded into the Argonaute-incorporated RNA-induced silencing complex (RISC) where, following the unwinding of the duplex, each mature miR strand, called guide, is retained. Then, the mature miR coordinates the RISC by partial complementarity between the miR and the mRNA target sequences. In this way, miRs can induce translational repression or mRNA destabilization and degradation.

MiR biogenesis is a complex process (Figure 2). Firstly, miRs are mainly transcribed by RNA polymerase II into primary transcript precursors (pri-miRNAs) that can range from hundreds to thousands nucleotides long. Next, pri-miRNAs are endonucleolytically cleaved in the nucleus by the RNases III Drosha and Pasha to generate ~70 nucleotides long hairpin pre-miRNAs. Then, the resulting precursors are actively exported from the nucleus to the cytoplasm by Exportin5, working with its cofactor Ran-GTP. In the cytoplasm, the RNase III Dicer recognizes the pre-miRNAs, cleaves off their loops and generates 20–22-nucleotides long miRNA duplexes characterized by two nucleotides protruding at each 3′-end. At this point, the functional strand of each mature miR is loaded into ribonucleoprotein complexes (miR-induced silencing complexes, miRISCs) together with the proteins from the Argonaute (Ago) family. Once loaded onto miRISC complexes, mRNAs may be silenced by cleavage, translational repression, or deadenylation [60].

MiRs may control several biological processes deregulated in cancer (differentiation, proliferation, and apoptosis), as well as the crosstalk between malignant cells and TME [63,64]. Therefore, miRs may behave as oncogenes or tumor suppressor genes, depending on the role of their targets. Generally, onco-miRs are gained in tumors and target tumor-suppressor genes and, vice versa, tumor suppressor miRs are downregulated or lost in cancer with the consequent upregulation of their oncogenic targets [65]. The first evidence of miR involvement in cancer goes back to the year 2002, when a frequent deletion of the miR-15a/16-1 cluster was found in patients with chronic lymphocytic leukemia (CLL), suggesting the “suppressive” role of this cluster [66]. After this first study, a great number of miRs have been found deregulated in various cancers. Notably, their role can vary based on the cell type (different kind of tumor or stromal cells) or on the context (different stage of the disease). It is emerging that miR deregulation is crucial not only in the cancer cell compartment, but also in stromal and immune cells.

Importantly, miRs are shuttled from cancer to stromal cells and vice versa through gap junctions or via EVs. In particular, miRs are the main cargo in exosomes, vescicles with 30–150 nm diameter. Several cells in TME, including TAMs, TECs, and ICs, are able to communicate thanks to EV secretion [63]. Interestingly, EVs and their cargoes may affect not only the surrounding cells (autocrine or paracrine effects), but they may travel through the vascular system reaching different organs where they prepare the pre-metastatic niche (endocrine effect). miRs are sorted into EVs by proteins such as hnRNPA2B1, Annexin A2, Y-box protein 1, and Ago2 and, in some cases, the changes in miR sorting into EVs are linked to tumor progression [67]. Notably, miRs delivered into target cells are functional and exert their activity through the same machinery and mechanisms used by endogenous miRs, resulting in the recipient cell modulation and reprogramming [68]. Melo et al. reported that exosomes derived from breast cancer cells contained pre-miRNAs and proteins of the RISC complex, thus resulting able to process pre-miRNAs into mature miRs [68].

From a translational point of view, miRs can be detected in up to 12 biological fluids including blood, urine, and saliva (in EV or EV-free) [69]. Several studies have shown the potential of miRs as biomarkers for cancer detection and/or prognosis [70]. In addition, miR inhibition, generally achieved by antisense oligonucleotides complementary to a specific miR (anti-miR oligonucleotides) can have a therapeutic value [71,72]. Examples are antagomirs and locked nucleic acids (LNAs). These modifications could be imposed on tumor cells as well as on the cells of the TME. In fact, it is becoming more and more evident that targeting or re-education of the TME could improve the outcome of the disease [6] and miRs could be potentially exploited for this purpose.

In the next paragraphs, the metabolic reprogramming mediated by miRs in different cell types of the TME is discussed and summarized in Table 1.

### 4.1. miRs in the Metabolic Crosstalk between Tumor Cells and CAFs

Among the TME cell components, the tumor-activated form of fibroblasts known as CAFs are the most abundant cells and they can be originated in different ways. They can derive from normal fibroblasts, following a change of features due to the crosstalk with cancer cells. Alternatively, CAFs can originate from bone-marrow-derived MSCs that during tumor development are recruited into the TME by inflammatory molecules such as the chemokines CCL5 and CXCL16. Once in the TME, MSCs can differentiate into endothelial precursors or into CAFs when they are stimulated by transforming growth factor β (TGF-β), stromal derived Factor-1 (SDF-1), or osteopontin [89]. Once CAFs have been generated, they secrete MMPs, cytokines, chemokines, growth factors and, therefore, they participate in the deposition and remodeling of the ECM and promote tumor progression as well as an immune escape [90]. Relevantly, CAFs are also actively involved in the metabolic rewiring of the TME [22] and this is, at least partially, mediated by miRs. Zhang and colleagues demonstrated that two induced-CAF models (TGF-β and PDGF-induced), as well as human CAFs isolated from colon cancers and melanomas, switch their metabolism from OXPHOS to aerobic glycolysis based on the intervention of the enzyme isocitrate dehydrogenase 3α (IDH3α) controlled, in turn, by miR-424 [73]. In fact, the reduced expression of IDH3α, due to increased levels of miR-424 in CAFs, impairs the ratio of α-ketoglutarate (α-KG) and succinate with a consequent downregulation of α-KG levels, thus leading to the inhibition of proline hydroxylase (PHD2) responsible of HIF-1α hydroxylation. As a result, HIF-1α becomes more stable and it promotes the uptake of glucose as well as the upregulation of glycolytic enzymes, causing increased glycolysis and inhibition of OXPHOS by upregulation of NDUFA4L2 (NDUFA4 mitochondrial complex associated like 2), the negative regulator of complex I [73].

As described above, miRs and, in particular, circulating miRs are crucial regulators of metabolism reprogramming during cancer progression. miRs can be exploited by cancer cells as messengers able to modify the metabolic profiling at the metastatic sites. Fong and colleagues [74] demonstrated, both in vitro and in vivo, that breast cancer cells secrete miR-122-rich EVs which are uptaken by the stromal cells present in the lung and brain metastatic niches. In these cells, miR-122 controls the expression of the pyruvate kinase reducing the glycolysis and glucose metabolism. As a result, glucose uptake by lung fibroblasts and astrocytes of the niches is reduced, leading to an increased availability of this nutrient to the invading malignant cells favoring the metastatization process. From a therapeutic point of view, miR-122 inhibition using antisense oligonucleotides reduces breast cancer metastasis to the brain and lungs in preclinical models via the inhibition of this metabolic remodeling, giving hope for therapeutic interventions.

miR-122 is not the only miR involved in tumor–stromal metabolic crosstalk, in fact, Yan and colleagues [75] identified miR-105 as a potent regulator of metabolic reprogramming in the TME. They demonstrated that breast cancer cells secrete EVs containing miR-105 whose expression is under the control of MYC in cancer cells and, once released, miR-105 is able to activate the MYC signaling in CAFs inducing metabolic reprogramming. CAFs shape their metabolic landscape based on the modifications occurring in the environment. In presence of high levels of nutrients, miR-105 induces glucose and glutamine metabolism to fuel surrounding cancer cells. Upon reduction of nutrients and increase of metabolic by-products, miR-105-reprogrammed CAFs start to detoxify from metabolic waste such as lactic acid and ammonium. Following these detoxification reactions, CAFs generate energy-rich metabolites, exploited by cancer cells.

In a melanoma setting, Shu and colleagues [76], investigated the relevance of local acidification of the stroma to favor pre-metastatic niche formation. The authors analyzed the ability of exosomes derived from melanoma cells in reprogramming dermal fibroblasts and they observed increased aerobic glycolysis and decreased OXPHOS, thus leading to increased extracellular acidification. When the content of EVs derived from six different melanoma cells, both BRAFV600E mutated and wild type was analyzed, it became evident that the presence of miR-155 and miR-210 was instrumental in promoting glycolysis and inhibiting OXPHOS. In fact, when the activity of miR-155 and miR-210 was blocked, the metabolic switch induced by melanoma EVs was reverted.

The relevance of the hypoxia-regulated miR-210 in metabolism is well-grounded. Hypoxic senescent fibroblasts foster prostate cancer aggressiveness by inducing EMT and by secreting energy-rich compounds to support tumor growth. Increased miR-210 expression in young fibroblasts promotes senescence-associated markers, as well as conversion into CAFs, as demonstrated by [77].

**Figure 3:**
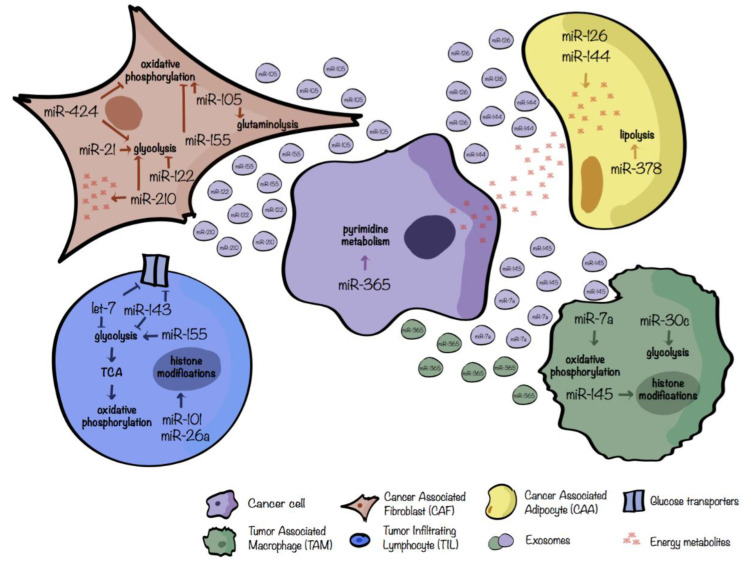
miRs involved in the regulation of tumor–stromal metabolic crosstalk. miRs involved in the metabolic crosstalk between tumor cells (**violet**); cancerassociated fibroblasts, CAFs (**brown**); cancerassociated adipocytes, CAAs (**yellow**); tumorinfiltrating lymphocytes, TILs (**blue**); tumorassociated macrophages, TAMs (**green**). Only the main metabolic pathways were taken into account and both secreted or differentially expressed miRs were considered. Extracellular vescicle color refers to the cell of origin.

In pancreatic cancer, miR-21 is upregulated in stroma, in particular in CAFs. Co-cultures of miR-21 overexpressing CAFs with pancreatic cancer cell lines promote tumor progression, whereas its downmodulation in CAFs inhibits glycolysis in these cells and disrupts the stroma-tumor metabolic crosstalk, thus preventing tumor progression [78]. A summary of the main miR players involved in the crosstalk between CAFs and tumor cells is shown in Figure 3.

### 4.2. miRs in the Metabolic Crosstalk between Tumor Cells and CAAs

Adipocytes are the most abundant components of the adipose/fat tissue which stores and mobilizes lipids, controlling the energy homeostasis of the organism [91]. For certain tumors, such as breast and prostate cancers, the TME is enriched of adipocytes, close to cancer cells or in direct contact with them, called cancerassociated adipocytes (CAAs) capable of secreting inflammatory factors, growth factors or cytokines that can trigger EMT, or releasing metabolites supporting cancer cell proliferation [79]. Wu et al. demonstrated [83] that upon human breast cancer cell interaction, adipocytes evolve towards a beige/brown phenotype and release different metabolites such as lactate, pyruvate, free fatty-acids (FFAs) and ketone bodies. Conversely, tumor cells exhibit metabolic adaptation following co-culturing with mature adipocytes. miRs are crucial regulators of this metabolic switch. In fact, exosomes obtained from co-cultures of tumor cells and adipocytes contain high levels of miR-144 and miR-126. miR-144 fosters beige/brown adipocyte features by downregulating the MAP3K8/ERK1/2/PPARγ pathway while miR-126 acts on the AMPK/autophagy pathway by disrupting IRS/Glut-4 signaling and stabilizing HIF-1α expression [79]. One of the main functions of adipocytes is the hydrolysis of lipid triglycerides, stored in the cytoplasmic lipid droplets, to fatty acid and glycerol, a metabolic process called lipolysis. Deregulation of lipolysis can contribute to cancer-associated cachexia which is a life-threating disorder characterized by loss of body weight due to reduced muscle and adipose tissue mass [93]. Cancer-cachexia is linked to increased chemotherapy toxicity, poor quality of life and increased mortality and, therefore, it is a serious medical problem [93]. To this regard, starting from a miR profiling on adipose tissue of cachexic versus weight-stable patients with gastrointestinal cancer, Kulyté et al. identified a significant upregulation of miR-378 in the adipose tissue of cachexic individuals. In vitro analyses revealed that miR-378 boosts lipolysis. Even if the precise mechanism and targets are not described, miR-378 can, indirectly, induce the expression of several key lipolytic-related genes such as the adipose triglyceride lipase PNPLA2, the tri/di-glyceride hormone-sensitive lipase (HSL) and the lipid droplet-coating protein Perilipin 1 (PLIN1). Overall, the increased lipolysis related to miR-378 expression could be involved in the decrease of adipose tissue loss and cancer cachexia [80]. Figure 3 depicts the crosstalk between tumor cells and adipocytes.

### 4.3. miRs in the Metabolic Crosstalk between Tumor Cells and TAMs

Among the most abundant ICs present in the TME, we can find TAMs [45]. Macrophages are a heterogeneous antigen-presenting population characterized by their ability to shape their phenotype depending on the surrounding environmental cues. They can range from a pro-inflammatory—anti-tumoral (M1-like) to an anti-inflammatory—wound healing—pro-tumoral (M2-like) phenotype [47], characterized by a more glycolytic or oxidative metabolism, respectively [7]. In the TME, macrophages are generally polarized into M2-like and they express high levels of anti-inflammatory cytokines, chemokines, growth factors, angiogenic factors, and proteases, creating an immunosuppressive TME, and promoting tumor progression and metastatic dissemination. Clinically, high density of TAMs usually, but not always, correlates with a poor prognosis in cancer patients [94,95].

In human gastric cancer, Zhihua and colleagues [88] found that miR-30c promotes glycolysis and, thus, M1 polarization by regulating the mTOR pathway in TAMs. Mechanistically, miR-30c targets the ‘Regulated in Development and DNA damage response 1’ (REDD1) gene, a negative regulator of mTOR, enhancing the PI3K/Akt/mTOR pathway. In a tumor context, the hypoxic environment reduces miR-30c levels, thus, decreasing the percentage of anti-tumoral M1 macrophages. Besides the direct effect of macrophage polarization, Wenes et al. [96] showed that highly glycolytic REDD1-deficient TAMs compete with endothelial cells for glucose usage. The result of this competition induces tumor vessel normalization and, consequently, prevents metastasis formation [96], indicating that besides a chemokine or protease mediated control of angiogenesis by TAMs, the competition for nutrients between TAMs and ECs plays an equally important role.

Immune and cancer cells cannot only metabolically influence each other by competing for the same substrates (such as glucose), but also by releasing miRs that regulate metabolic pathways. For example, in pancreatic adenocarcinoma, Binenbaum et al. [82] showed that miR-enriched exosomes are released by TAMs and uptaken by cancer cells where they induce resistance to gemcitabine (a cytidine analogue) due to an alteration of cancer metabolism. Even if the precise mechanism still needs to be elucidated, miR-365, among all the miRs, plays a major role in this process by upregulating the pyrimidine metabolism and, thus, increasing the triphosphate-nucleotide pool that competes with gemcitabine for the incorporation into the DNA of cancer cells. Moreover, miR-365 also induces an upregulation of cytidine deaminase (CDA), the enzyme that catabolizes gemcitabine.

Cancer cells develop several mechanisms to modulate the TME in order to elicit immune escape as a part of a process called immunoediting. Among all these mechanisms, several publications involve tumor-derived exosomes as critical immunosuppressive mediators [97]. In this regard, Park et al. [83] showed that hypoxic cancer cells, such as melanoma cells, release miR-7a-enriched exosomes triggering M2-like polarization of TAMs through, at least partially, their metabolic reprogramming. Indeed, miR-7a suppresses several target genes of the insulin pathway such as INS-1 and IGF1R and, thus, probably reduces the PI3K/Akt/mTOR signaling pathway resulting in an increased OXPHOS and, in turn, M2 polarization. Similarly, miR-145 is enriched in EVs derived from colorectal cancer cells and induces the pro-tumoral, M2-like polarization, typical of TAMs [84]. In this case, miR-145 regulates the histone deacetylase HDAC11 increasing the acetylation of histone H3, thus favoring, for example, the expression of the anti-inflammatory cytokine IL-10. Figure 3 sums-up the crosstalk between macrophages and cancer cells.

### 4.4. miRs in the Metabolic Crosstalk between Tumor Cells and TILs

Tumor Infiltrating Lymphocytes (TILs) are involved in the antigen-specific responses against tumors. They are activated, upon priming, by the tumor antigens presented directly by cancer cells or by antigen-presenting cells (APCs), such as macrophages and DCs [98].

T cells constitute the majority of TILs and can be further subdivided in several helper CD4^+^ T cell subsets (Th1, Th2, Th17, Tregs) and in cytotoxic CD8^+^ T cells. Regarding CD4^+^ T cells, Th1 helper cells secrete pro-inflammatory cytokines favoring immune response while Th2, Th17, and Tregs mediate immunosuppression [22]. Cytotoxic T cells are considered one of the most powerful anti-tumoral ICs, but they are often absent or dysfunctional, due to their exhaustion or to the immunosuppressive features of the TME [11]. They exert anti-tumoral activity by directly killing malignant cells through the release of granules of perforin and granzyme B. Clinically, high levels of T cells correlate with good prognosis in several cancers—such as melanoma, breast, lung, ovarian, renal, prostate, and gastric [99]—and in recent years, immunotherapy approaches have been developed to foster the anti-tumoral capacity of T cells [85].

Metabolically, naïve T cells, that are the T cells that have not yet encountered their cognate antigen, rely mainly on OXPHOS while activated cytotoxic CD8^+^ T cells switch to aerobic glycolysis [48]. In this regard, Wells and colleagues [96] identified let-7 miRs as key modulators for the maintenance of naïve phenotype of CD8^+^ T cells by modulating T cell metabolism. They found that let-7 regulates, in addition to proliferation and differentiation, glycolysis, and protein synthesis by reducing, likely via MYC, the transcriptional levels of key glycolytic enzymes (Gpd2, Pfk1, Hk2, Tpi, Pkm, and Ldha), glucose transporters (Glut1, Glut2) and the protein synthesis enzyme Yars. Upon T cell activation, let-7 levels are reduced, MYC is derepressed and the metabolic switch from OXPHOS to glycolysis occurs in order to obtain a proper cytotoxic T cell response towards virus-infected cells or antigen-loaded cancer cells [85]. MiR-155 is one of the most studied miRs involved in immune response and, specifically, in T cell response where it regulates cytokine and interferon signaling through STAT1 [100] and SOCS1 [101]. Recently, miR-155 has also been linked to T cell metabolism. In particular, Monnot and colleagues found that, through the targeting of the inositol 5-phospatase Ship1, an inhibitor of the mTOR pathway, miR-155 promotes mTOR activity and, consequently, CD8^+^ T cell glycolysis, increasing T-cell proliferation and effector functions [86]. Using OVA-expressing melanoma models, it has been shown that miR-155 exogenous overexpression in antigen-specific CD8^+^ T cells improves their anti-tumoral activity against low-affinity antigens [86]. Overall, these results reinforce the findings of Dudda and colleagues that indicate how the over-expression of miR-155 might improve T cell adoptive-transfer therapy in cancer as well as in infectious diseases [101].

Importantly, after the primary immune response, a small portion of T cells gives rise to antigen-specific long-lived T-cells, named T-memory, which respond better and faster to a second antigen exposure [98]. From a metabolic point of view, memory CD8^+^ T cells and CD4^+^ Tregs rely more on enhanced OXPHOS and lipid oxidation [48] rather than glycolysis as done by activated cytotoxic CD8^+^ T cells. Zhang et al. [87] showed that miR-143 promotes central T memory differentiation by suppressing glycolysis through the modulation of GLUT1. Interestingly, the immunosuppressive metabolite IDO and its product kyneureine, generally produced in the TME [102], downregulate miR-143 levels highlighting the bidirectional link between miR-143 and metabolism.

In the TME, cancer cells compete with activated T cells for the consumption of resources such as glucose, necessary to fuel anaerobic glycolysis. Ultimately, tumor-imposed glucose restriction mediates a reduction of anti-tumoral T cell activity leading to tumor progression [43,103]. Interestingly, also miRs play a role in reducing CD8^+^ T cell functions in a glucose restricted microenvironment. Indeed, glucose shortage mediated by cancer cells increases miR-101 and miR-26a levels in CD8^+^ T cells, leading to the reduction of their common target, the methyltransferase EZH2, a key enzyme of the epigenetic polycomb repressive complex 2 (PRC2). In turn, reduced activity of EZH2 dampens anti-tumoral CD8^+^ T cell responses favoring immune subversion [103]. A summary of the main miR players involved in TILs-tumor cell crosstalk is shown in Figure 3.

## 5. Conclusions and Future Perspectives

Growing evidence supports the old scattered observations reporting that cancer cells display a peculiar metabolism, therefore, underlying the relevance of metabolic alterations for cancer progression [14,104]. In parallel, we need to consider a tumor as an entity formed not only by proliferating malignant cells, but embedded in a complex TME where ECM, stroma, and ICs are present and constantly modified and reshaped in order to support tumor growth and metastasis dissemination (Figure 1) [4,103]. Even if the crosstalk between malignant and non-malignant cells of the primary tumor mass has already been well studied in terms of growth factors and cytokines [105,106], the relevance of certain metabolites and substrate competition in the crosstalk between cancer cells and stroma or immune cells remains to be unraveled. In addition, it is still not clear how cancer cells can induce the metabolic reprogramming of the surrounding cells of the TME and which mechanisms take place in the stroma and ICs. From the studies discussed in this review, it becomes apparent that, by directly modulating metabolic enzymes, transporters, and important regulators of metabolic processes, miRs, could play an important role in mediating the metabolic reprogramming in the TME. Of course, the field is in continuous evolution and many aspects still remain to be elucidated. Many studies, leading to important discoveries summarized in this review, were performed using simplified in vitro models in which only single interactions between cancer and stromal/immune cells were analyzed instead of considering the complex tumor mass in which metabolite composition and oxygen tension can be quite different (hypoxic conditions or nutrient deprivation). Overall, there is an urgent need to improve models in order to better understand tumor–stroma metabolic crosstalk as in the real tumor context. From the therapeutic point of view, metabolic reprogramming represents a new window of opportunity for fighting cancer. Various studies highlight drug repurposing of metabolic-based drugs in a cancer setting [107]. Notably, miRs could be involved in the effects mediated by these drugs and in chemoresistance. For instance, metformin, the most used drug in type-II diabetes, controlling glucose uptake and gluconeogenesis, induces the expression of the tumor suppressor miRs let-7 and miR-26 in breast [108], colorectal [109], pancreatic [110], oral [111], and renal [112] cancers. In this line, drugs acting on de novo fatty acid synthesis influence miR expression levels. Simvastatin increases let-7 expression by decreasing NF-kB and lin-28b [113]. This evidence suggests that metabolic-based drugs could target miRs involved in the communication between tumor and TME, giving hope for new therapeutic interventions.

## Figures and Tables

**Table 1:** MiRs involved in tumor–stroma metabolic crosstalk

miR	Position	TME Component	Cancer	Targets /Pathways	Metabolic Pathway	Ref
miR-424	Xq26.3	CAFs	melanoma	IDH3a	oxidative phosporylation	[73]
miR-122	18q21.31	CAFs	breast	PKM2	glycolysis	[74]
miR-105	Xq28	CAFs	breast	MYC	glutaminolysis/oxidative ohosphorylation	[75]
miR-155	21q21.3	CAFs	melanoma	n/a	oxidative phosporylation	[76]
miR-210	11p15.5	CAFs	melanoma	n/a	glycolysis	[76]
miR-210	11p15.5	CAFs	prostate	n/a	energy rich compounds	[77]
miR-21	17q23.2	CAFs	pancreatic	n/a	glycolysis	[78]
miR-144	17q11.2	CAAs	breast	MAP3K/ERK1/2/PPARg	glycolysis	[79]
miR-126	9q34.3	CAAs	breast	AMPK	glycolysis	[79]
miR-378	5q32	CAAs	gastrointestinal cancer	n/a	lipoylisis	[80]
miR-30c	6q13	TAMs	gastric cancer	REDD1	glycolysis	[81]
miR-365	16p13.12	TAMs	pancreatic	CDA	pyrimidine metabolism (in cancer cells)	[82]
miR-7a	9q21.32	TAMs	melanoma	insulin-Akt-mTOR	oxidative phosporylation	[83]
miR-145	5q32	TAMs	colorectal cancer	HDAC11	histon acetylation	[84]
let-7	9q22.32	TILs	mastocytoma	MYC EOMES	glycolysis	[85]
miR-155	21q21.3	TILs	melanoma	INPP5D	glycolysis	[86]
miR-143	5q32	TILs	esophagus	GLUT1	glycolysis	[87]
miR-101	1p31.3 9p21.1	TILs	ovarian	EZH2	epigenetic modifications	[88]
miR-26a	3p22.2	TILs	ovarian	EZH2	epigenetic modifications	[88]

## Data Availability

Not applicable.
